# Roles of FcRn in Antigen-Presenting Cells during Autoimmunity and a Clinical Evaluation of Efgartigimod as an FcRn Blocker

**DOI:** 10.3390/pathogens12060817

**Published:** 2023-06-08

**Authors:** Yihan Zhou, Shisong Jiang

**Affiliations:** Department of Oncology, University of Oxford, Old Road Campus Research Building, Roosevelt Drive, Headington, Oxford OX3 7DQ, UK; yihan.zhou@oncology.ox.ac.uk

**Keywords:** antigen-presenting cells, neonatal crystallizable fragment receptor (FcRn), FcRn blockade, efgartigimod, autoimmune diseases, autoimmune therapeutics

## Abstract

The immune system is a complex network of multiple cells, tissues, and organs that protects the body against foreign pathogenic invaders. However, the immune system may mistakenly attack healthy cells and tissues due to the cross-reactivity of anti-pathogen immunity, leading to autoimmunity by autoreactive T cells and/or autoantibody-secreting B cells. Autoantibodies can accumulate, resulting in tissue or organ damage. The neonatal crystallizable fragment receptor (FcRn) is an important factor in immune regulation through controlling the trafficking and recycling of immunoglobulin G (IgG) molecules, the most abundant antibody in humoral immunity. In addition to its role in IgG trafficking and recycling, FcRn is also involved in antigen presentation, which is a crucial step in the activation of the adaptive immune response via directing the internalization and trafficking of antigen-bound IgG immune complexes into compartments of degradation and presentation in antigen-presenting cells. Efgartigimod, an FcRn inhibitor, has shown promise in reducing the levels of autoantibodies and alleviating the autoimmune severity of myasthenia gravis, primary immune thrombocytopenia, and pemphigus vulgaris/foliaceus. This article aims to provide an overview of the importance of FcRn in antigen-presenting cells and its potential as a therapeutic target in autoimmune diseases, using efgartigimod as an example.

## 1. Introduction

The process of immune homeostasis is essential for human survival. It protects us from foreign pathogenic invasion while maintaining self-tolerance [1]. Both innate and adaptive immune systems play critical roles in protecting against foreign invaders, including microorganisms and viruses. The recognition of pathogen-associated molecular patterns (PAMPs) and damage-associated molecular patterns (DAMPs) by pattern recognition receptors (PRRs) on innate immune cells triggers the activation of innate immune responses [2,3]. Among these responses, antigen-presenting cells (APCs) are stimulated and are responsible for presenting antigens to adaptive immune cells, B cells and T cells [4,5].

However, some viral infections, for instance, herpes, rubella and measles, can dysregulate immunity and lead to the onset of autoimmune diseases due to antigenic molecular mimicry, epitope spreading and/or other mechanisms [6]. In these cases, the antiviral immunity cross-reacts with self-antigens, leading to the accumulation of activated autoreactive B or T cells [6,7,8]. Autoantibodies or autoreactive T cells can serve as indicative biomarkers for autoimmunity. Autoimmune diseases are rare, inter-individually distinct, and underreported. They remain a leading cause of death in young and middle-aged females [9]. The annual increase in autoimmune incidence and prevalence calls for urgent action to develop effective interventions to lower the level of autoantibody titers and/or autoimmune cell activity [10].

**Figure 1:**
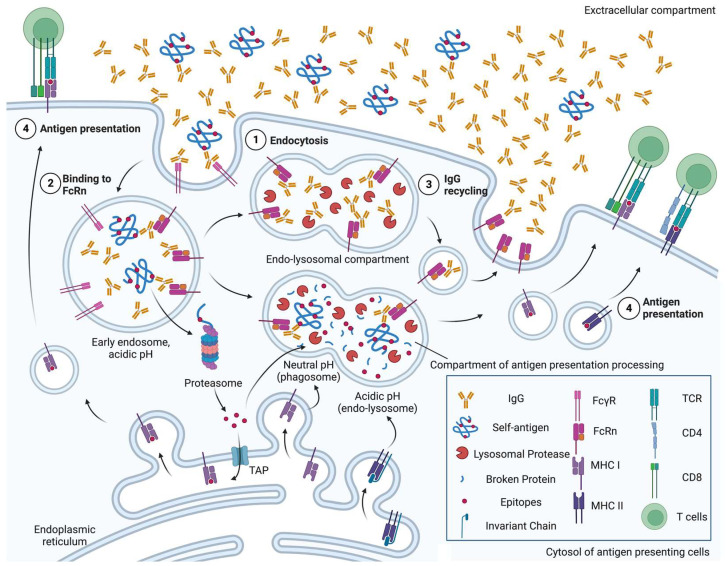
A schematic overview of neonatal crystallizable fragment receptor (FcRn)-mediated roles of antigen-presenting cells in autoimmunity. ① Immunoglobulin G (IgGs), regardless of binding to self-antigen, are endocytosed by either pinocytosis or under mediation by receptors, e.g., Fc-gamma receptor (FcγR). ② In the early endosomes, where pH is acidic, FcRn can bind to both IgGs and the immune complex of IgG and self-antigen. ③ FcRn-bound IgGs are not degraded by lysosomal proteases, and are recycled back to the cell surface. ④ FcRn preserves self-antigen by binding to IgGs in the acidic endosome, so that self-antigens can be exported into the cytosol later for proteasomal degradation, imported into major histocompatibility complex (MHC) class I molecule-containing endoplasmic reticulum via transporter of antigen presentation (TAP) and cross-presented, in the phagosome-to-cytosol pathway. Otherwise, FcRn traffics IgG-bound self-antigen into compartments for degradation at a neutral pH (phagosome) or acidic pH (endo-lysosome) to generate epitopes that are presented on MHC class I molecules, in the vacuolar pathway, or MHC class II molecules, respectively [18]. Figure created with Biorender.com (accessed 2 June 2023) (Agreement no. XI25FUGU8I).

Several interventions have been applied to lower the disease-causing levels of autoantibodies, including plasma exchange [11] and intravenous (IV) immunoglobulin [12]. Recent studies have shown that inhibiting the neonatal crystallizable fragment receptor (FcRn), a receptor for the crystallizable fragment (Fc) of immunoglobulin G (IgG), can effectively reduce the level of IgGs and alleviate the severity of humoral autoimmune disorders such as myasthenia gravis [13], experimental ulcerative colitis [14], chronic inflammatory demyelinating neuropathy [15] and primary immune thrombocytopenia [16]. As summarised in Figure 1, FcRn also mediates antigen presentation and activates T cell immunity, highlighting its pathological potential in T-cell-based autoimmunity [17].

This article aims to provide an overview of the immunological significance of FcRn expressed on APCs and highlight its potential as a therapeutic target against autoimmune diseases. Using efgartigimod as an example, we present clinical evidence that indicates FcRn inhibition as a powerful tool for the treatment of autoimmune diseases.

## 2. IgG, FcRn and Their Interaction in Contributions to Humoral Autoimmunity

Antibodies, including autoantibodies, are glycoproteins that play a crucial role in immune and immunopathological responses [19]. They are categorized into five different isotypes: monomers IgD, IgE or IgG, dimer IgA or pentamer IgM. Among them, IgM is the first isotype expressed by immature B cells, and the level of IgD is then seen in association with the differential activation stage of naïve B cells [20]. At the secondary lymphoid organs, B cells can also serve as APCs, presenting antigens internalized via the B cell receptor (BCR) onto major histocompatibility complex (MHC) class II molecules to the T cell receptor (TCR) of CD4+ helper T cells. This is followed by the costimulatory signal of CD40-CD40L binding and cytokine secretion of interleukin (IL)-4, leading to B cell activation and antibody class switching to other three isotypes, such as IgM being switched to IgG [21].

IgG is the most abundant and life-long antibody circulating in the blood, and includes four highly conserved subclasses: IgG1, IgG2, IgG3 and IgG4 [22]. Each IgG, like other Ig isotypes, consists of two domains: the fragment of antigen binding (Fab) region, and the Fc region [23]. The Fab regions of antibodies bind to antigens to form immune complexes (ICs) that can be further endocytosed or trafficked via the binding of their Fc regions to their receptors [24].

Fc-gamma receptor (FcγR) is a class of receptors that bind to immunoglobulin G (IgG) antibodies, promoting their phagocytosis irrespective of the Fab region’s antigen-binding specificity [17]. In addition to FcγR, there is another atypical IgG-binding receptor called the neonatal crystallizable fragment receptor (FcRn) [24,25]. This receptor was first discovered by Brambell and is primarily involved in establishing essential passive immunity in neonates of rodents by transferring IgGs present in the mother’s milk into the neonatal bloodstream through the intestinal epithelial barrier [25].

In humans, FcRn is mainly expressed in the placenta to transport maternal IgG antibodies to the developing foetus. Over time, our understanding of FcRn has expanded, and it is present in various cell types, such as endothelial cells, epithelial cells and antigen-presenting cells such as dendritic cells, macrophages and B cells. FcRn is encoded by the *FCRGT* gene (Gene ID: 2217), located on chromosome 19q13.33, and is an MHC class I-like chain, which is non-covalently bound to β2-microglobulin [17,26,27]. Unlike other FcγRs, which require glycosylation at the Fc region [28,29], FcRn directly binds to IgG at residues Ile253, His310 and His435, which are well-conserved among IgG subclasses [17].

FcRn can regulate the intracellular trafficking and recycling of IgG molecules [30]. This receptor is predominantly located intracellularly, and has been shown to interact with IgG molecules in a pH-dependent manner. When IgG molecules are taken up by APCs through fluid-phase pinocytosis or receptor-mediated phagocytosis, they are internalized into early endosomes. In these compartments, FcRn interacts with IgGs at their Fc regions in a slightly acidic environment (pH 6.0–6.5) [31,32]. This interaction prevents IgGs from being degraded by lysosomes (pH 4.5–5.0), and instead recycles them back into the extracellular compartment at near-neutral pH (pH 7.4) [33,34].

Through this FcRn-mediated recycling process, IgGs can maintain high concentrations in the bloodstream, extending their half-life and increasing their effectiveness in combating viral and other pathogenic infections [23]. In contrast, other subclasses such as IgA or IgM are not recycled and are therefore more short-lived. This recycling mechanism is particularly important for IgG1 and IgG3, which have longer half-lives than IgG2 and IgG4. In summary, the interaction between FcRn and IgG molecules is essential for maintaining an effective IgG-mediated immune response.

The accumulation of pathogenic IgGs can contribute to the development of numerous autoimmune diseases. This is often caused by an overactive humoral immune response, as a potential result of antigenic mimicry or epitope spreading from anti-viral or microorganism immunity. The US National Health and Nutrition Examination Survey (NHANES) [35] has identified the detection of four autoantibodies in serum related to rheumatoid arthritis, Hashimoto’s thyroiditis or celiac disease. In individuals aged above sixty, at least one type of pathogenic autoantibody was detected in 12,800,000 individuals in the US. Pathogenic IgGs, such as anti-thyroperoxidase (TPO) antibodies in Hashimoto’s thyroiditis, can bind to TPO-expressing thyroid cells and intensify antibody-dependent cell-mediated cytotoxicity (ADCC) via Fc recognition by FcγRs on natural killer cells [36]. The increasing understanding of IgG-recycling FcRn has led to attempts to block FcRn, which can aid in disrupting the maintenance of high IgG levels under autoimmune conditions and alleviate the health burden associated with IgG-mediated diseases.

## 3. Antigen Presentation and T-Cell-Based Autoimmunity Mediated by FcRn-IC Binding

In addition to monomeric IgGs, FcRn can also mediate the internalization and trafficking of antigen-bound IgG immune complexes (Ag-IgG ICs) in APCs. These large complexes require receptors such as FcγRs to be taken up by APCs at near-neutral pH, which mainly interact with the ICs at the extracellular level [37]. Due to the high expression of FcRn in APCs, the immune functions of APCs are facilitated through the subcellular delivery of ICs to lysosomes, where degradation of opsonized antigens can occur, or to compartments where antigens are processed and presented via either MHC class I or II pathways, thereby extending the activation of adaptive immune responses [17].

### 3.1. MHC Class II Pathway

Recent studies have shown that FcRn not only mediates the recycling of monomeric IgGs, but also plays a critical role in trafficking Ag-IgG ICs. The invariant chain of MHC class II molecules can interact with and traffic FcRn through intracellular compartments [38], suggesting that FcRn can direct Ag-IgG ICs to the endo-lysosomal compartment for antigen degradation and then to the MHC class II compartment to present antigenic peptides and activate CD4+ T cell immune responses [39]. Several studies have provided evidence to support this hypothesis, as described below.

Végh et al. [40] used ovalbumin (OVA) as the antigen and found that bovine FcRn overexpression in transgenic (Tg) BALB/c mice promoted dendritic cell and B cell proliferation, increased the phagocytotic activity of dendritic cells fourfold, and led to a greater amount of OVA-IgG ICs, resulting in a sixfold proliferation increase in CD4+ T cells extracted from OVA_323-339_-specific, MHC class II (I-A^d^)-restricted TCR Tg DO11.10 mice.

Another study [41] designed the 4-hydroxy-3-ido-5-nitrophenylacetyl (NIP)-specific human IgG1 (^NIP^hIgG1) that forms ICs with NIP-conjugated antigens, including OVA (NIP-OVA), keyhole limpet hemocyanin (KLH) (NIP-KLH) or gliadin (NIP-gliadin). The study also designed the ^NIP^hIgG1 bearing amino acid substitutions from Ile253 to Ala253, His310 to Ala310 and His435 to Ala435 at the Fc region (IHH mutations) to disenable its binding with FcRn only. In vitro, murine BALB/c dendritic cells deficient in FcRn or loaded with IHH-mutated NIP-OVA-^NIP^hIgG1 ICs showed a reduction in the MHC class II presentation of OVA_323-339_ epitope and CD4+ DO11.10 T cell proliferation. Consistently, robust IL-2 secretion and T cell proliferation were observed in C57BL/6 mice in vivo after the injection of dendritic cells previously loaded with FcRn-binding NIP-KLH-^NIP^hIgG1 ICs, instead of those with FcRn deficiency or loaded with NIP-KLH alone or IHH-mutated NIP-KLH-^NIP^hIgG1 ICs. Human monocyte-derived dendritic cells presented FcRn-binding NIP-gliadin-^NIP^hIgG1 ICs more effectively to intestinal T cells of celiac disease patients than IHH-mutated NIP-gliadin-^NIP^hIgG1 ICs, which strengthens the essential role of FcRn in binding Ag-IgG ICs for their antigen presentation and T cell activation. The study also showed that FcRn co-localized with lysosomes, which are essential components of MHC class II antigen presentation, in human monocyte-derived dendritic cells with the presence of multimeric NIP-OVA-^NIP^hIgG1 ICs, whereas such co-localization was not observed with IHH-mutated NIP-OVA-^NIP^hIgG1 ICs or monomeric IgGs [41].

### 3.2. MHC Class I Pathway or Cross-Presentation

Dendritic cells play a critical role in the immune response by presenting exogenous antigens on MHC class I molecules through the process of cross-presentation. This process is particularly significant because it allows for the activation of CD8+ T cells, which are crucial in generating an effective immune response. CD8+CD11b- dendritic cells in mice [42] and CD141+(BCDA-3+) dendritic cells in humans [43] are considered the most efficient at cross-presentations of soluble antigens due to their neutral endosomal pH, which preserves antigens to generate MHC class I epitopes [44]. Two pathways for phagosomal cross-presentation have been proposed. The first involves the trafficking of antigens into phagosomes, where cathepsin S degrades them at a neutral pH before loading them onto MHC class I molecules. The second pathway suggests that antigens are preserved and exported into the cytosol, where they are degraded by proteasomes and then loaded onto MHC class I molecules [45].

Recently, Baker et al. [37] demonstrated that FcRn is essential for the cross-presentation of NIP-OVA in the form of NIP-specific IgG (NIP-OVA-^NIP^IgG) ICs to activate CD8+ OT-I (OVA_257-264_-specific, MHC class I (H-2k^b^)-restricted TCR Tg) T cells. They found that CD8-CD11B+ dendritic cells from C57BL/6 mice were particularly effective at cross-presentation, even at low doses of NIP-OVA-^NIP^IgG ICs. The presence of FcRn compensates for phagosomal acidification, allowing Ag-IgG ICs to bind to FcRn and be preserved similarly to antigens in the neutral endosomal compartment of CD8+CD11b- dendritic cells. The authors supported that FcRn mediates cross-presentation through the phagosome-to-cytosol pathway, involving proteasome and Sec61α. FcRn-mediated cross-presentation was only observed in infiltrating CD8-D11B++CD11C+ dendritic cells from C57BL/6 mice with chronic colitis, and not in FcRn-deficient Tg mice. This suggests that FcRn can mediate cross-presentation by CD8-D11B++CD11C+ dendritic cells to elicit CD8+ T cell immunity in vivo, even under the condition of colitis, potentially aggravating the auto-inflammatory symptoms via cellular autoimmunity.

Additionally, a recent study established that FcRn, acting as the coreceptor to FcγRIIa (CD32a), is a potential driver for autoimmunity [46]. The study demonstrated that FcRn can promote cross-presentation independently of FcγR and increase the secretion of cytokines, including TNF-α, IL-6, IL-12 and interferon (IFN)-γ, by CD8+ T cells in response to NIP-OVA-^NIP^hIgG1 ICs. These findings suggest that FcRn is a key mediator of phagosomal cross-presentation and has the potential to drive autoimmunity.

## 4. An FcRn Blocker, Efgartigimod, as a Therapeutic Strategy for Autoimmunity

In APCs, FcRn binds to the Fc regions of monomeric IgG molecules or Ag-IgG immune complexes (ICs) at an acidic pH, facilitating their transportation between different subcellular compartments [17]. FcRn plays a crucial role in maintaining humoral autoimmunity by transporting autoreactive IgGs back to the cell surface at an extracellular neutral pH, allowing them to circulate in the bloodstream [30]. Through FcRn-mediated transcytosis, self-reactive Ag-IgG ICs are delivered to the endo-lysosomal or phagosomal compartment of APCs, where the self-antigens in ICs can be processed to generate epitopes for presentation on MHC class I or II molecules. This process stimulates the activity of autoreactive CD8+ and CD4+ T cells, respectively [17]. The combination of high levels of autoantibodies and subsequent ADCC, along with CD4+ T cell activity, can further augment humoral immunity by promoting the differentiation of B cells into autoantibody-secreting plasma cells and memory cells, thus leading to excessive immune responses [39].

Given the pathogenic significance of FcRn in the humoral and cellular pathways of the adaptive immune system, it represents a novel therapeutic target [30]. FcRn inhibitors, such as efgartigimod, have been developed and are currently under clinical assessment for the treatment of myasthenia gravis, primary immune thrombocytopenia and pemphigus vulgaris/foliaceus. These inhibitors have shown promise in clinical trials, as they can not only accelerate the clearance of pathogenic IgGs and IgG-mediated autoimmunity [30], but also reduce the presentation of self-antigens to T cells, thereby decreasing the risk of self-tissue or organ damage [17]. Table 1 provides further details on completed clinical trials of efgartigimod.

Efgartigimod (VYVGAART^TM^ or ARGX-113) is a novel, first-in-class therapeutic agent that targets FcRn and has been approved by the FDA for the treatment of IgG-mediated myasthenia gravis [47]. It is a human Fc fragment derived from the IgG1 antibody, and consists of 227 amino acids, which form two identical chains linked by two disulfide bonds that give it its high affinity for FcRn. Unlike the native human IgG1 Fc fragment, efgartigimod is optimized with MST-HN mutations (from Met252 to Tyr252, Ser254 to Thr254, Thr256 to Glu256, His433 to Lys433 and Asn434 to Phe434), which enhance its affinity for binding FcRn in different pH environments, either on the near-neutral cell surface or in acidic subcellular compartments, and outcompete the binding of FcRn to endogenous pathogenic IgGs [48].

Once efgartigimod binds to FcRn, the unbound endogenous IgGs are excluded from the recycling and are subsequently degraded by lysosomes, leading to a decrease in IgG levels and regulating IgG-mediated autoimmune destruction. A first-in-human study [48] has shown that efgartigimod can reduce IgG levels by up to 50% at maximum at a single dosage of 10 to 50 mg/kg (*n* = 4 vs. 2 treated with placebo, per cohort) and by approximately 75% at multiple dosages of 10 mg/kg (*n* = 6 vs. 2 treated with placebo, per cohort) compared to baseline. Despite the full recovery of IgG levels eight weeks after the last dose, efgartigimod has shown promising results in regulating IgG levels, thus alleviating IgG-mediated autoimmune diseases.

**Table 1:** A list of completed clinical trials of efgartigimod in myasthenia gravis, primary immune thrombocytopenia and pemphigus vulgaris/foliaceus with data from [49,50,51,52,53].

Disease	Phase	Number	Eligibility	Enrolment	Treatment	Outcome
Myasthenia gravis	II	NCT02965573	≥18 years old; MGFA Class II–IVa; positive for anti-AChR; ≥5 points higher and ≥50% nonocular attributable to MG-ADL; stable standard-of-care	24	Four weekly 10 mg/kg IV administrations	Safety, efficacy, pharmacology and immunogenicity
	III	NCT03669588	≥18 years old; MGFA Class II–IV, ≥5 points higher and ≥50% nonocular attributable to MG-ADL; stable standard-of-care	167; 129 being positive for anti-AChR	Four weekly 10 mg/kg IV administrations per cycle; ≥8-week cycle interval up to three cycles	Safety, efficacy, pharmacodynamics and immunogenicity
Primary immune thrombocytopenia	II	NCT03102593	<30 × 10^9^/L average of two platelet count; no single >35 × 10^9^/L; stable standard-of-care	38	Four weekly 5 or 10 mg/kg IV administrations	Safety, efficacy, pharmacology and immunogenicity
	III	NCT04225156	<30 × 10^9^/L average of two platelet count; no single >35 × 10^9^/L; stable standard-of-care	131	Four weekly 10 mg/kg IV administrations; follow-up of weekly or biweekly dosing	Efficacy, safety and pharmacodynamics
Pemphigus vulgaris/foliaceus	II	NCT03334058	<45 PDAI; positive for Dsg-1/3 or IgG deposits on keratinocyte surface; discontinuity of immunosuppression	34 for safety test; 31 for efficacy test	Four weekly 10 or 25 mg/kg IV administrations; follow-up of IDMC recommended dosing	Safety, efficacy, pharmacology and immunogenicity

Abbreviations: AChR, acetylcholine receptor; Dsg, desmoglein; IDMC, Independent Data Monitoring Committee; IV, intravenous; MG-ADL, Myasthenia Gravis Activities of Daily Living; MGFA, Myasthenia Gravis Foundation of America; PDAI, Pemphigus Disease Area Index.

### 4.1. Efgartigimod in Myasthenia Gravis

In patients with myasthenia gravis, the majority (80 to 85%) have anti-acetylcholine receptor (AChR) antibodies (IgG1 and IgG3) [54,55], while the remainder have autoantibodies that target other components of the neuromuscular junction, such as anti-muscle-specific kinase IgG4 and anti-lipoprotein-receptor related protein 4 IgG1 or IgG3 [56,57]. The efficacy of efgartigimod in treating generalized myasthenia gravis has been evaluated in several clinical trials, including an exploratory phase II study (NCT02965573) [49], a multi-center, placebo-controlled, double-blinded, randomized phase III trial (ADAPT) (NCT03669588) [50] and an ongoing open-label extension (NCT03770403). In the phase II study [49], patients (*n* = 12 vs. 12 treated with placebo) were given four 10 mg/kg IV administrations of efgartigimod, and the treatment was found to dramatically expedite the clearance of IgGs, as evidenced by a reduction in the level of total serum IgGs and anti-AChR antibodies by 40 to 70% compared with the baseline, in the first or second week following the last dose. Simultaneously, patients treated with efgartigimod showed a rapid response and persistent clinical improvement, with statistically significant reductions in Myasthenia Gravis Activities of Daily Living (MG-ADL) scores, Quantitative Myasthenia Gravis (QMG) scores, Myasthenia Gravis Composite (MGC) scores and 15-item Quality of Life scale for Myasthenia Gravis (MG-QoL15r) scores compared with the placebo treatment. The effect of efgartigimod was even maintained two weeks after the last administration, with 75% of patients still showing significant improvements in MG-ADL score. Most adverse events were mild or unrelated to the treatment, with no withdrawal due to death or severe toxicity compared with the placebo treatment. The ongoing open-label extension trial will provide further insight into the long-term safety and efficacy of efgartigimod in treating myasthenia gravis patients. Results from this trial are expected to be revealed in June 2023.

In the phase III ADAPT study [50], efgartigimod was found to be well-tolerated and effective in treating myasthenia gravis patients, particularly those with high levels of anti-AChR antibodies. The study investigated up to three treatment cycles, with four weekly IV administrations of 10 mg/kg efgartigimod given each cycle. The primary endpoint, MG-ADL responders (≥2-point and ≥4-week reduction in MG-ADL score), was achieved by 68% of 65 anti-AChR antibody positive patients treated with the first cycle of efgartigimod compared to 30% of the 64 treated with placebo (*p* < 0.0001), increasing to 78% after subsequent cycles. A similar 68% (vs. 37% treated with placebo, *p* < 0.0001) was observed in all patients (*n* = 84 vs. 83 treated with placebo). QMC responders (≥3-point and ≥4-week reduction in QMC score) after the first cycle made up 63% (vs. 14% treated with placebo, *p* < 0.0001) of anti-AChR antibody-positive patients. In addition, the maximum score improvement from the baseline in four clinical efficacy factors, MG-ADL, QMG, MCG, and MG-QOL15r, was achieved one or two weeks after the first cycle, with high proportions showing improvement. These results demonstrate that efgartigimod is a potent drug that provides clinical benefits for myasthenia gravis patients, particularly those with high levels of anti-AChR antibodies.

### 4.2. Efgartigimod in Primary Immune Thrombocytopenia

Primary immune thrombocytopenia is a bleeding disorder characterized by a low circulating platelet count (<100 × 10^9^/L), which is mostly caused by the recognition of platelet-associated glycoprotein (GP) [58] by IgG antibodies. To investigate the efficacy of efgartigimod in treating primary immune thrombocytopenia, Newland et al. [51] conducted a randomized, placebo-controlled, double-blinded phase II trial (NCT03102593) involving patients with an average platelet count of less than 30 × 10^9^/L and a single count of less than 35 × 10^9^/L. In the trial, patients were administered four weekly IV doses of either efgartigimod (*n* = 13 at 5 mg/kg and *n* = 12 at 10 mg/kg) or placebo (*n* = 13). Both doses of efgartigimod resulted in an immediate reduction in overall IgG levels by 60.4% and 63.7%, respectively, with no change in IgG levels observed in the placebo group. Moreover, more than 40% of patients treated with efgartigimod demonstrated a reduction in at least one of the anti-platelet IgGs against GPIIB/IIIa, GPIbI/X or GPIa/IIa, with mostly mild-to-moderate toxicity. After efgartigimod administration, an identical proportion of patients treated with either 5 or 10 mg/kg achieved a platelet count of ≥50 × 10^9^/L (53.8%) compared to 50% of patients treated with placebo. In addition, 46.2% or 38.5% of patients treated with 5 or 10 mg/kg of efgartigimod, respectively, achieved a platelet count of ≥100 × 10^9^/L, compared to only 8.3% of patients treated with placebo. Furthermore, 38.5% of efgartigimod-treated patients achieved either an International Working Group (IWG)-defined response (≥2-fold increase and ≥7-day platelet count between ≥30 × 10^9^/L and < 100 × 10^9^/L) or a complete response (≥2-fold increase and ≥7-day platelet count ≥100 × 10^9^/L), which lasted for 21 weeks after efgartigimod administrations. The mean cumulative duration for platelet count ≥50 × 10^9^/L was 24.5 days (vs. 7.3 days treated with placebo), and 38.5% of efgartigimod-treated patients reached > 10-day cumulative duration. In addition, the proportion of bleeding incidence, as assessed by the immune thrombocytopenia-specific bleeding assessment tool (ITP-BAT) using the World Health Organization (WHO) standard, decreased by 46.2% and 38.5% in the 5 and 10 mg/kg efgartigimod-treated populations, respectively, compared to 33.3% in the placebo-treated population. Overall, the results of the trial suggest that efgartigimod is an effective treatment option for primary immune thrombocytopenia patients.

In the ADVANCE IV trial (NCT04188379) [52], a multi-centre, placebo-controlled, randomized phase III clinical investigation, pre-treated patients (*n* = 86 vs. 45 treated with placebo) with an average of two platelet counts <30 × 10^9^/L received four weekly administrations of 10 mg/kg efgartigimod, followed by response-dependent regimens of either weekly or biweekly dosing for the next 20 weeks. An immediate reduction in IgG by 60% from the baseline was observed, with serious adverse events discovered in 8.1% (vs. 15.6% treated with placebo) of the efgartigimod-treated population. The platelet response was sustained in 21.8% (vs. 5.0% treated with placebo, *p* < 0.05) of patients (*n* = 78 vs. 40 treated with placebo) diagnosed with chronic immune thrombocytopenia and 25.6% (vs. 6.7% treated with placebo, *p* < 0.05) of all patients after efgartigimod treatment. Furthermore, 51.2% of the efgartigimod-treated population (vs. 20.0% treated with placebo) presented the International Working Group (IWG)-defined response, highlighting the potential of efgartigimod to rescue the IgG-mediated loss of platelets and potentially reverse the pathogenesis of primary immune thrombocytopenia. An open-label extended trial (ADVANCE +) (NCT04225156) has been initiated to further investigate the efficacy and safety of efgartigimod in this patient population.

### 4.3. Efgartigimod in Pemphigus Vulgaris/Foliaceus

Pemphigus foliaceus is a skin-limited autoimmune disease caused by a dysfunctional immune system that attacks desmoglein (Dsg)-1, the inter-keratinocyte adhesive junction in the epidermal layer, predominantly with IgG4 subclasses [59]. On the other hand, pemphigus vulgaris is the most common form of the disease, affecting both cutaneous and mucosal areas due to Dsg-1 and -3, respectively [60]. A phase II trial (NCT03334058) [53] followed by a phase III placebo-controlled, double-blinded, randomized trial (NCT04598451) evaluated the efficacy (*n* = 16) and safety (*n* = 19) of efgartigimod in mild pemphigus vulgaris/foliaceus patients. The patients were given four weekly IV 10 mg/kg efgartigimod administrations (*n* = 19 for the safety test and 16 for the efficacy test) as induction therapy, followed by maintenance dosing regimens according to the Independent Data Monitoring Committee (IDMC)’s recommendations, or 25 mg/kg (*n* = 15) each week continuously until the end of consolidation (≥80% lesion recovery and ≥2-week free of new lesions) up to 34 weeks. Efgartigimod was well-tolerated, and a reduction of 50% in the median Pemphigus Disease Area Index (PDAI) score was observed after 10 mg/kg induction or a reduction of 52% after 25 mg/kg induction. Disease control was achieved in 90% of the overall population during induction, with no relapses observed during or after maintenance. After induction, a median 61% reduction in the level of anti-Dsg-1 antibodies and a 49% reduction in anti-Dsg-3 antibodies was observed, and the reduction was sustained at 70% and 42%, respectively. Although the overall IgG levels returned to baseline after the termination of administration, the sustained reduction in pathogenic anti-Dsg-1 and anti-Dsg-3 antibodies suggests that efgartigimod could be effective in controlling the severity of pemphigus by selectively regulating pathogenic IgGs.

## 5. Conclusions and Future Directions

FcRn is associated with both IgG-recycling and antigen presentation in APCs [17,30], which can contribute to autoimmune pathogenesis. Inhibiting FcRn can reduce T cell activation and autoantibody-mediated immunity. However, the mechanism by which FcRn distinguishes between IgGs and Ag-IgG ICs that are directed specifically into compartments of antigen presentation is not clear. FcRn has been shown to aid cross-presentation in non-professional cross-presenting dendritic cells, and guide the trafficking of Ag-IgG ICs into the phagosomal compartment containing MHC class I molecules in professional cross-presenting cells [37,46]. However, more research is needed to understand the implications of the FcRn-mediated cross-presentation and its comprehensive contribution to auto-inflammation and autoimmunity.

The FcRn blockage with efgartigimod is a therapeutic approach that has been shown to increase the degradation of IgGs and improve the health and quality of life of patients. Its pharmacokinetics are linear and well-tolerated, with mostly mild-to-moderate or unrelated adverse events. Compared to other treatments, it is a fast-acting and short-term therapeutic approach that can be carefully monitored for personalization via different multiple dosing strategies. Further studies should be conducted to identify the subset of activated T cells under FcRn immune regulation and potential disease-contributing molecular pathways to precisely define the specific pathology of each autoimmune disease and the therapeutic universality of the FcRn blockage. Since FcRn blockers would be restricted to IgG-associated autoimmune diseases, patients should be screened for pathological associations with IgGs before clinically using FcRn inhibitory strategies. Nevertheless, the FcRn blockage certainly introduces therapeutic possibilities for other autoantibody- and/or T-cell-mediated autoimmune disorders, such as chronic inflammatory demyelinating polyneuropathy, myositis and multifocal motor neuropathy, which have been included in future studies by the efgartigimod company, argenx [61]. It is also noteworthy that the self-administrated subcutaneous treatment of FcRn blockers can potentially leverage the benefits and convenience, as long as clinical evidence for safety profiling is provided.

Overall, FcRn can facilitate both humoral and cellular immunity, and this fact makes FcRn a powerful target in therapeutic development for autoimmune diseases. An in-depth investigation into mechanisms underlying the FcRn regulation of APC functions, and consequently autoimmune pathology, will be an important future direction that should contribute to the therapeutic use of FcRn blockage agents.

## Data Availability

Not applicable.
